# Retrospective Cohort Analysis of the Relationship Between Emergency Department Length of Stay and Timing of First Laboratory Orders

**DOI:** 10.7759/cureus.68966

**Published:** 2024-09-08

**Authors:** Wayne A Martini, Nicole R Hodgson

**Affiliations:** 1 Emergency Medicine, Mayo Clinic, Phoenix, USA

**Keywords:** diagnostic timing, ed operations, emergency department, healthcare efficiency, laboratory orders, length of stay, patient management, patient throughput, physician age, retrospective analysis

## Abstract

Background

The efficiency of patient management in the Emergency Department (ED) is critical for optimizing healthcare delivery. Provider in triage (PIT) and similar ED flow models attempt to expedite throughput by decreasing the amount of time between patient arrival and initial order placement. The exact relationship between ED length of stay (LOS) and the timing of the first laboratory order, however, is unclear. The varying speed at which clinicians of different ages place laboratory orders and move patients through an ED also is understudied.

Methods

A retrospective analysis was conducted using SQL from the Clarity data archive to pull all patient encounters in 2023. Linear regression models using Analysis ToolPak in Microsoft Excel were used to create and examine the relationship between LOS and the timing of the first laboratory order. Secondary outcomes using the same models were created to analyze the impact of clinician age on LOS and the relationship between clinician age and the timing of first laboratory orders.

Results

Two hundred sixty-nine thousand eight hundred and eight ED visits were reviewed across three academic and 17 community emergency departments. We report a weak but statistically significant positive relationship between the timing of the first laboratory order and LOS (R² = 0.0378, p < 0.001). Secondary outcomes indicated a very weak negative correlation between clinician age and LOS (R² ≈ 0, p < 0.001) and no significant relationship between clinician age and the timing of the first laboratory order (R² ≈ 0, p > 0.05).

Conclusion

The timing of the first laboratory order is a significant, albeit weak, predictor of LOS in the ED. Clinician age has minimal impact on LOS and does not significantly influence the timing of the first laboratory order.

## Introduction

Efficient patient throughput and resource utilization in the Emergency Department (ED) are crucial for maintaining high-quality healthcare delivery, patient satisfaction, and healthcare sustainability [1]. The length of stay (LOS) in the ED is a key operational metric that reflects the efficiency of care provided. Various factors have been hypothesized to influence LOS, including the timing of diagnostic tests such as laboratory orders. Timely initiation of these tests is believed to be critical in reducing LOS, thereby improving patient throughput and overall ED efficiency.

In addition to the timing of laboratory orders, the characteristics of the clinician managing patients may also play a role in ED operations. Specifically, clinician age, often correlated with clinical experience, could potentially impact decision-making processes and workflow efficiency [2]. Previous research has suggested that more experienced clinicians may have different approaches to patient care, which could influence how quickly they initiate diagnostic orders and manage patient flow. However, the impact of clinician age on specific operational metrics, such as the timing of diagnostic orders and LOS, remains largely understudied. Understanding whether clinician age is a significant factor in ED efficiency is important for informing workforce planning and training initiatives.

The issue of prolonged LOS is further exacerbated by the phenomenon of boarding, where patients are kept in the ED while waiting for inpatient beds. Boarding, where patients are kept in the ED while waiting for inpatient beds, leads to overcrowded conditions, strain on resources, and can compromise patient privacy and the quality of care provided [1,3-5]. Patients who experience extended LOS in the ED are at increased risk for complications such as hospital-acquired infections and delays in receiving necessary treatments. Additionally, long LOS can negatively impact the overall throughput of the ED, reducing the department's capacity to treat new patients requiring emergency care. Therefore, addressing the factors that contribute to extended LOS is critical for improving ED efficiency, patient outcomes, and satisfaction [6].

There is substantial evidence indicating that delayed diagnostic testing and treatment initiation are significant contributors to extended LOS in EDs [7]. By optimizing the timing of these interventions, significant improvements in patient throughput and satisfaction may be achieved. Investigating the specific impact of lab order timing on LOS offers valuable insights into how ED operations can be enhanced to better serve patients.

This study aims to elucidate the relationship between LOS and the timing of the first laboratory order in the ED and to identify opportunities to improve efficiency, enhance patient perception of care quality, and reduce boarding without compromising patient care. Additionally, we investigate the effect of clinician age on LOS and the timing of the first laboratory order, hypothesizing that clinician age may influence these operational metrics due to variations in clinical decision-making and experience.

## Materials and methods

Study design and population 

This retrospective cohort study was conducted across Mayo Clinic, a large healthcare system with 17 community sites and three large academic centers in Minnesota, Arizona, Florida, Iowa, and Wisconsin in the United States from January 1, 2023, through December 31, 2023. The study analyzed data from all ED visits, capturing a broad spectrum of patient demographics and clinical scenarios, reflecting real-world conditions.

Sample size calculation 

Due to the large dataset comprising 269,808 ED visits, a formal sample size calculation was not required. Instead, the analysis focused on ensuring that the study had sufficient statistical power to detect meaningful effects, which was inherently achieved given the volume of data.

To ensure that the sample size was adequate for detecting significant relationships, an initial power analysis was conducted using a hypothesized small effect size (Cohen's f^2 = 0.02) with an alpha level of 0.05 and a power of 0.80. This power analysis suggested that a minimum sample size of 10,000 patients would be required, which was far exceeded by the final sample size.

Sampling technique 

A consecutive sampling technique was employed, where all eligible ED visits during the study period were included. This approach minimized selection bias and ensured that the sample was representative of the typical patient flow and case mix in the participating EDs.

Patient population

Inclusion Criteria

The study included all 269,808 ED visits within the specified dates across all participating sites who had at least one lab ordered during their visit to the department.

Exclusion Criteria

The study excluded patients who left against medical advice or left without being seen.

Data collection 

Data were extracted from the hospital’s electronic health record (EHR) system, including demographic information, lab order times, and LOS. The EHR system, Epic, allowed for detailed and accurate tracking of patient interactions within the ED, capturing timestamps for various events, including arrival, lab orders, and discharge. Additionally, the clinician's date of birth was recorded in the system allowing for accurate calculation of the age of the provider at the time of encounter. 

Data analysis

Descriptive statistics were used to summarize the study population, including means, medians, and standard deviations for continuous variables, and frequencies and percentages for categorical variables. The primary analysis involved linear regression models to evaluate the relationships between the timing of the first lab order and ED LOS. Secondary analyses examined the impact of clinician age on LOS and the timing of lab orders.

Statistical analysis 

Primary Outcome Analysis

The relationship between the timing of the first lab order and LOS was assessed using simple linear regression, with the timing of the first lab order as the independent variable, and LOS as the dependent variable.

Secondary Outcome Analyses

The impact of clinician age on LOS was analyzed using a separate linear regression model, with LOS as the dependent variable and clinician age as the independent variable. Another linear regression model was used to assess the relationship between clinician age and the timing of the first lab order.

A p-value of < 0.05 was considered statistically significant. All analyses were performed using Analysis Toolpak in Microsoft Excel, part of Microsoft Office 365.

Institutional Review Board (IRB) approval 

This study was approved by the Institutional Review Board (IRB) at Mayo Clinic, under approval number 24-008835. The IRB determined that the study posed minimal risk to participants and waived the requirement for informed consent due to the retrospective nature of the study and the use of de-identified data.

## Results

Table 1 provides a comprehensive overview of the baseline characteristics of the study population, detailing the age distribution, biological sex, race, and ethnicity of the 269,808 patients included in the analysis. The median LOS was 167 minutes, with an interquartile range (IQR) of 193 minutes. The median time to the first laboratory order was 12.9 minutes, with an IQR of 34.1 minutes

**Table 1:** Baseline characteristics of the study population.

Characteristic	Total (N = 269,808)
Age, Years	
Mean (SD)	54.16 (23.78)
Median (IQR)	58 (39)
Biologic Sex, n (%)	
Male	122,000 (45.22%)
Female	147,767 (54.77%)
Nonbinary	25 (0.01%)
Unknown	16 (0.01%)
Race, n (%)	
American Indian or Alaska Native	1,938 (0.72%)
Asian	5,806 (2.15%)
Black or African American	14,957 (5.54%)
Native Hawaiian or Pacific Islander	600 (0.22%)
White	239,206 (88.65%)
Unknown or Not Reported	7,301 (2.71%)
Ethnicity, n (%)	
Hispanic or Latino	17,759 (6.58%)
Not Hispanic or Latino	246,651 (91.44%)
Unknown or Not Reported	5,398 (2.00%)

Primary outcome: length of stay and timing of first lab order

**Figure 1:**
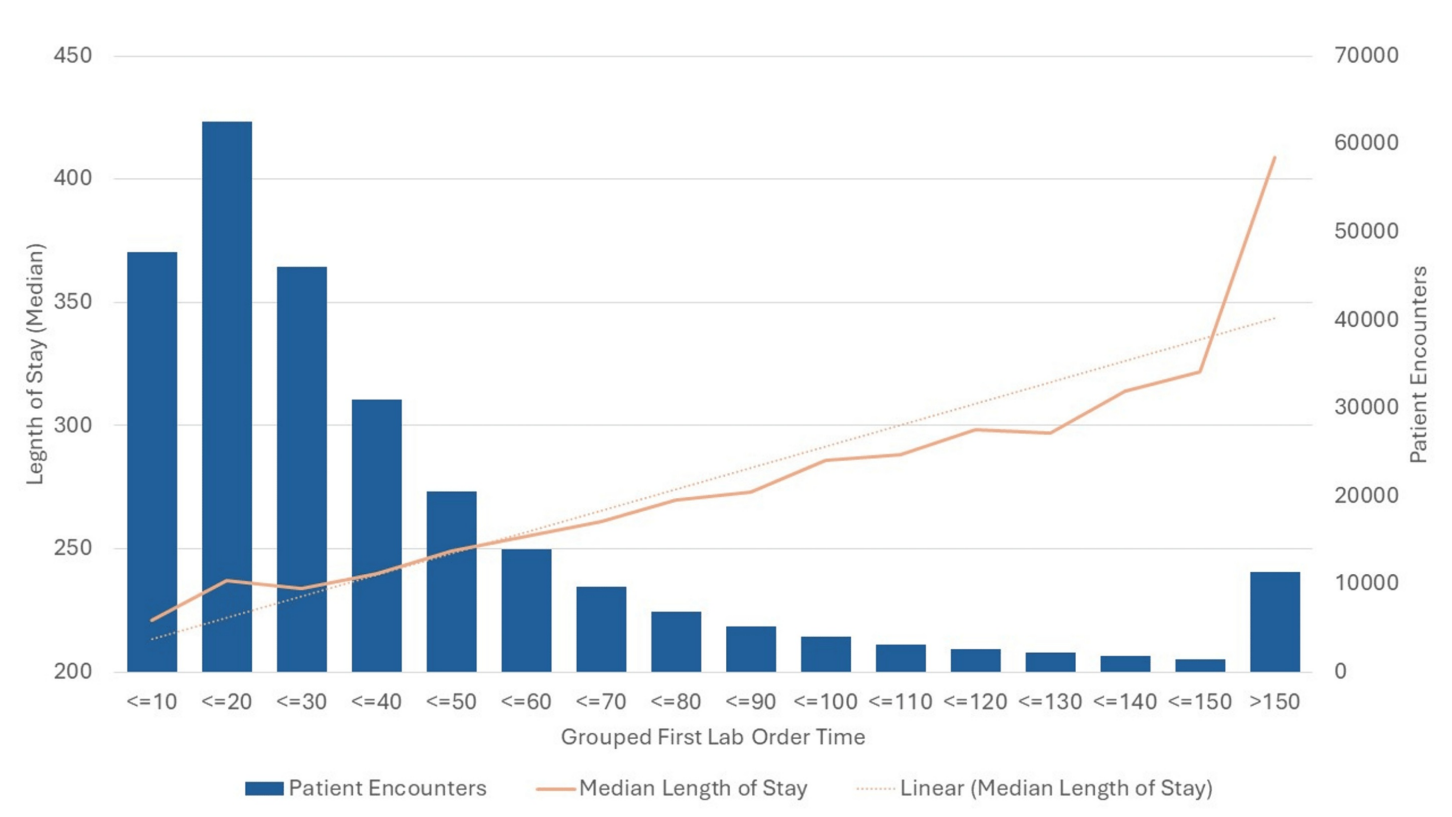
Graph comparison of length of stay vs grouped first lab order time.

The regression analysis revealed a weak but statistically significant positive relationship between the timing of the first lab order and LOS (R² = 0.0378, p < 0.001) (Table 2). This suggests that delays in ordering the first laboratory test are associated with longer stays in the ED (Figure 1). Specifically, for every minute of delay in ordering lab tests, the length of stay increases by approximately 18.6 seconds (calculated from a coefficient of 0.31 minutes * 60 seconds/minute = 18.6 seconds).

**Table 2:** Summary of regression analysis for primary and secondary outcomes LOS: length of stay.

Variable	Coefficient	Standard Error	t-Statistic	p-Value	R^2^
Time of First Lab Order	0.31	0.003	102.89	< 0.001	0.0378
Clinician Age and LOS	-0.72	0.20	-3.66	< 0.001	0.00005
Clinician Age and First Lab Order	0.02	0.12	0.13	0.893	0.000000067

Secondary outcome: clinician age and length of stay

**Figure 2:**
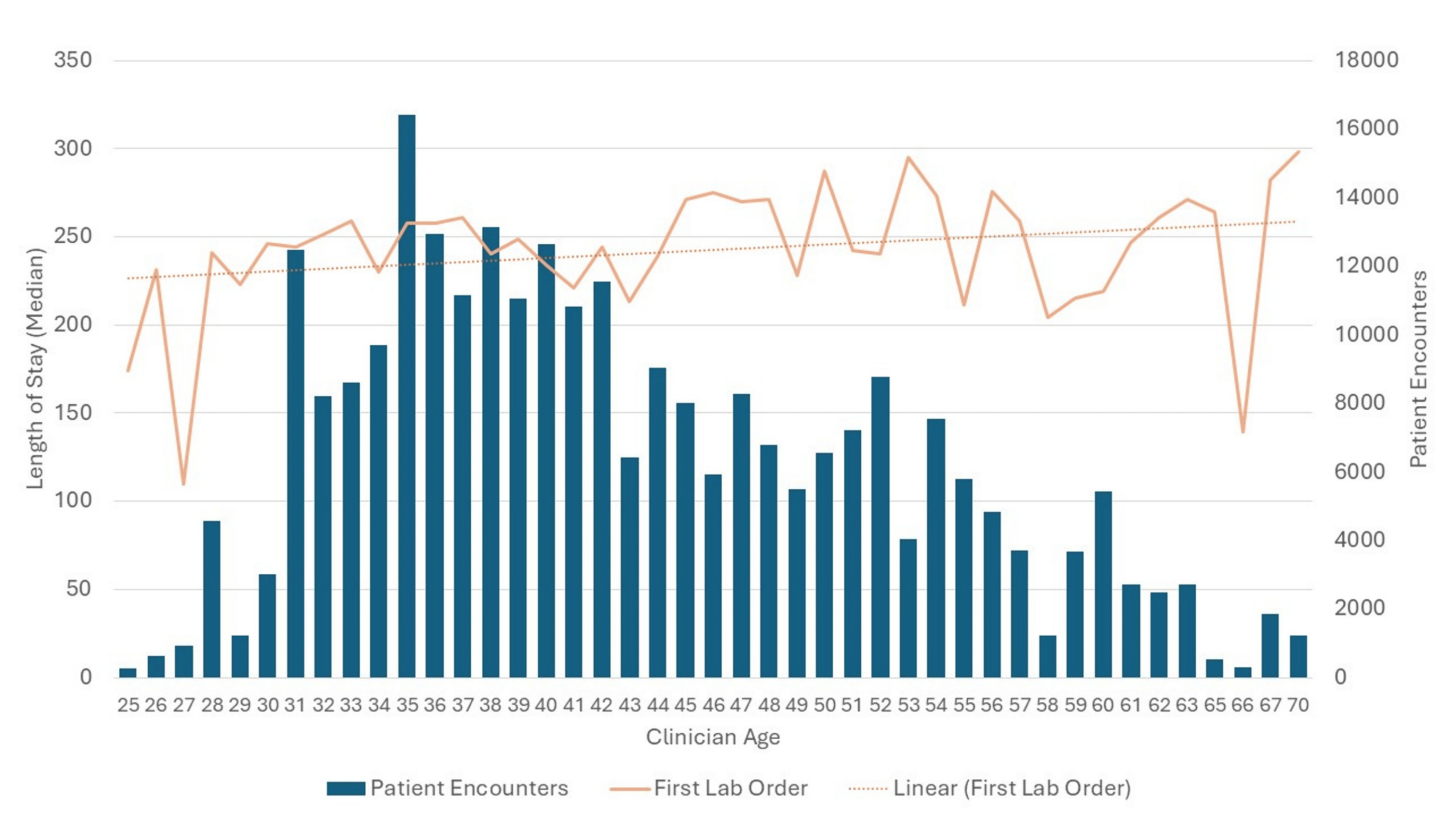
Graphical representation of data showing comparison of clinician age graphed against length of stay. Although statistically significant, the effect size is negligible, indicating that clinician age has minimal impact on LOS.

The analysis of clinician age and LOS showed a very weak negative correlation, with an R² value close to 0 (p < 0.001) (Table 2). This means that clinician age explains almost none of the variability in LOS, suggesting that knowing a clinician’s age does little to predict a patient's length of stay. While the correlation is statistically significant, it is so weak that the relationship between clinician age and LOS is not strong or practically meaningful (Figure 2). Other factors play a much larger role in the length of stay.

Secondary outcome: clinician age and timing of first lab order

**Figure 3:**
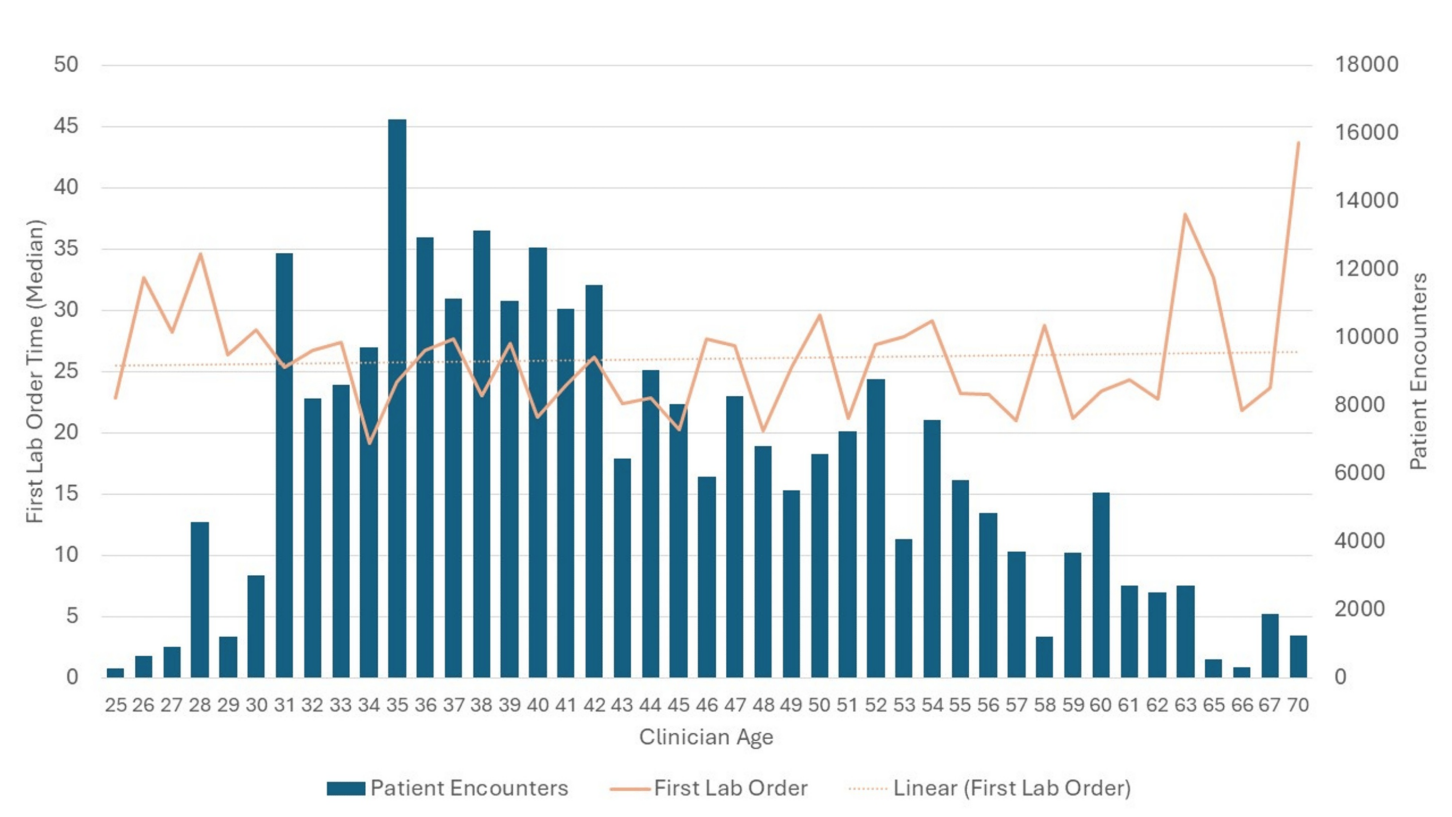
Graphical representation of relationship between clinician age and timing of first lab orders. There was no significant relationship between clinician age and the timing of first lab orders (R² ≈ 0, p > 0.05), suggesting that the age of the clinician does not influence when the first lab order is placed.

The analysis of clinician age and median first-order time showed a very weak negative correlation, with an R² value close to 0 (p < 0.001) (Table 2). This means that clinician age explains almost none of the variability in median first lab order time, suggesting that knowing a clinician’s age does little to predict the time of the first lab order being placed (Figure 3). While the correlation is statistically significant, it is so weak that the relationship between clinician age and lab order placement time is not strong or practically meaningful.

## Discussion

The findings of this study suggest that while the timing of the first lab order significantly impacts the LOS in the ED, the effect is weak. For every minute that the patient is present in the ED without labs ordered, it increases the length of stay by 18 seconds. This weak association indicates that other factors are likely to contribute more substantially to LOS. Secondary outcomes demonstrate that clinician age has negligible effects on both LOS and the timing of lab orders, suggesting that age-related factors of clinicians do not substantially influence these aspects of patient care.

Delays in ordering the first laboratory tests in the ED can be attributed to several interrelated factors that impact workflow efficiency and patient care timelines. One primary reason is the high patient volume and corresponding clinician workload, which can overwhelm available resources and lead to prolonged initial assessments and decision-making processes [8]. Additionally, complexities in patient presentations, particularly in cases with unclear symptoms or multiple comorbidities, may necessitate extended evaluation periods before appropriate lab tests are identified and ordered [9]. Inefficient triage systems and delays in initial patient assessments further contribute to postponements in lab ordering, as patients may not be promptly prioritized based on the severity of their conditions [10]. Operational issues such as limited availability of support staff, suboptimal electronic health record (EHR) interfaces, and lack of standardized protocols for common clinical presentations can also impede the timely initiation of necessary laboratory investigations [11,12].

Addressing these delays requires a multifaceted approach focused on optimizing ED processes and resource allocation. Implementing streamlined and standardized protocols or order sets for frequent clinical scenarios can expedite decision-making and reduce variability in care [13]. Enhancing triage efficiency through the adoption of models like provider in triage (PIT) ensures earlier clinical evaluation and faster initiation of appropriate diagnostic testing [14]. Adequate staffing levels, including both clinical and support personnel, are essential to manage high patient volumes effectively and distribute workload evenly among team members [15]. Improving EHR systems by customizing interfaces and incorporating quick-order functionalities can facilitate faster and more intuitive ordering processes for clinicians [16]. Ongoing training and education programs aimed at enhancing clinicians' familiarity with protocols and EHR usage can further reduce delays in lab ordering. Collectively, these strategies can contribute to more timely diagnostic evaluations, reduced ED length of stay, and improved overall patient outcomes.

Larger contributors to LOS

Several other factors likely have a more significant influence on LOS in the ED. These include patient acuity, staffing levels, availability of diagnostic resources, and hospital bed capacity [17-21].

Patients with higher acuity and more complex medical conditions require more comprehensive evaluations and treatments, which naturally extend their LOS [22]. These patients often need multiple diagnostic tests, consultations with specialists, and extended periods of monitoring, all of which contribute to longer stays in the ED [23].

The number and efficiency of ED staff, including clinicians, nurses, and support personnel, significantly impact LOS [21,24]. Adequate staffing ensures that patients are assessed and treated promptly, while efficient workflows can reduce delays in care delivery. Conversely, understaffed or overburdened EDs may experience bottlenecks that extend patient stays [7].

The availability and turnaround time of diagnostic resources, such as imaging services and laboratory tests, are critical determinants of LOS [23,25]. Delays in obtaining and processing these tests can lead to prolonged waiting times for patients. Streamlining diagnostic processes and enhancing resource availability can therefore help reduce LOS [6].

One of the major contributors to extended LOS in the ED is the availability of inpatient beds [17]. Patients who require admission may remain in the ED for extended periods if there are no available beds in the hospital. This boarding phenomenon not only increases the LOS for these patients but also impacts overall ED throughput by occupying critical space and resources [1].

Implications for practice 

Given the multifactorial nature of LOS in the ED, efforts to reduce LOS should adopt a holistic approach. This includes ensuring that EDs and labs are adequately staffed with well-trained personnel can improve the efficiency of patient care and reduce bottlenecks [18,26]. Streamlining the ordering, processing, and reporting of diagnostic tests can help minimize delays. Additional investing in point-of-care testing and advanced imaging technologies has been proven to reduce LOS [21,27,28]. Additionally, patient workup complexity increases LOS when considering when to use either IV or oral contrast for CT imaging [25,29].

Implementing protocols and pathways for common conditions can standardize care and reduce variability in LOS. Rapid assessment and triage protocols can ensure that high-acuity patients receive timely care [3]. Hospitals should work on strategies to reduce boarding by improving inpatient bed management and discharge processes. This can involve coordinating with other hospital departments to ensure timely transfers and admissions [30].

Study strengths and limitations 

The retrospective design limits the ability to infer causality. The very high number of patient encounters (269,808) across different sites and practice environments (three academic, 17 community) significantly strengthens the hypothesis. By including both community sites and academic centers, we were able to ensure that the findings would be applicable across various types of EDs, from smaller, local hospitals to large, tertiary care centers. Potential confounders, such as patient comorbidities and the severity of presenting complaints, were not fully accounted for, which may influence the results. Clinician experience was unable to be accurately ascertained from the medical record; ergo, age was used as a proxy. While our analysis reviewed the clinician's age and time of the first laboratory order, it does not imply there is no effect of clinician experience on the time of the first laboratory order. Additionally, variability in data recording practices and accuracy in the EHR system may affect the reliability of the findings. Departmental variability such as the use of clinicians in triage at some sites may also impact results.

Future research 

Further research is needed to identify and address the other major contributors to LOS in the ED. Prospective studies that can control for a broader range of variables and incorporate more detailed clinical data will be valuable. Additionally, exploring interventions that target multiple aspects of ED operations simultaneously may provide insights into more effective strategies for reducing LOS.

## Conclusions

The timing of the first laboratory order is a weak predictor of LOS in the ED. The timing of lab orders has a statistically significant association with LOS, showing for every minute that passes without lab placed, LOS increases by approximately 18.6 seconds. Clinician age does not significantly impact LOS or the timing of first lab orders.
